# Metabolic health is more closely associated with prevalence of cardiovascular diseases or stroke than obesity: A cross-sectional study in Korean populations

**DOI:** 10.1097/MD.0000000000003902

**Published:** 2016-06-17

**Authors:** A Ri Byun, Seungwon Kwon, Sang Wha Lee, Kyung Won Shim, Hong Soo Lee

**Affiliations:** a Department of Family Medicine, School of Medicine, Ewha Womans University, Seoul, Korea; b Department of Cardiology and Neurology, College of Korean Medicine, Kyung Hee University, Seoul, Korea.

**Keywords:** cardiovascular disease, metabolically healthy obese, obesity, risk factor, stroke prevalence

## Abstract

Mounting evidence suggests that not all obese subjects are at increased cardiovascular risk. However, the relationship between the metabolically healthy obese (MHO) phenotype and cardiovascular diseases (CVDs) or stroke remains unclear. Therefore, we retrospectively investigated the prevalence of CVDs or stroke according to metabolic health with obese.

We studied 3695 subjects (40–85 years) from the Fifth Korean National Health and Nutrition Examination Survey. Participants were divided into 2 groups and 6 subgroups based on the body mass index (BMI) and metabolic syndrome (MetS) components: healthy (exhibiting none of the 5 MetS components) with the followings: healthy-normal weight (BMI < 23 kg/m^2^), healthy-overweight (BMI = 23–24.9 kg/m^2^), and healthy-obese (BMI ≥ 25 kg/m^2^); and unhealthy (exhibiting 2 or more MetS components) with the followings: unhealthy-normal weight, unhealthy-overweight, and unhealthy-obese.

In the healthy group (n = 1726), there were 76 CVDs or stroke patients (4.4%), whereas in the unhealthy group (n = 1969), there were 170 (8.6%). The prevalence was significantly different between the 2 groups (*P* *<* 0.001). However, the prevalence was not significantly different among healthy subgroups (*P* = 0.4072). The prevalence in unhealthy subgroups also demonstrated no statistically significant difference (*P* = 0.3798).

We suggest that the prevalence of CVDs or stroke is different between metabolically healthy and unhealthy phenotype. Furthermore, MHO did not reveal higher CVDs or stroke prevalence rather than metabolically healthy other groups. Additional cohort studies are needed to explain causality between CVDs or stroke incidence and subjects exhibiting the MHO phenotype.

## 1 Introduction

The prevalence of obesity is increasing worldwide, with the condition predicted to affect >1 billion people by 2030.^[1]^ The growing obesity epidemic is associated with a sharp increase in obesity-related cardiovascular diseases (CVDs) such as hypertension and type 2 diabetes mellitus, and consequently increases the risk of all-cause mortality, as well as that of coronary artery disease and CVDs mortality.^[2–4]^ However, mounting evidence suggests that not all obese subjects are at increased cardiovascular risk; the “metabolically healthy obese” (MHO) phenotype may exist in the absence of metabolic abnormalities.^[5]^ MHO is characterized by its low abundance of metabolic abnormalities such as insulin resistance, proatherogenic lipoprotein profile, pro-inflammatory state, or hypertension. In addition, they present with less visceral and hepatic muscle fat accumulation, and fewer gene expression-encoding markers of adipose cell differentiation.^[6,7]^ Despite long-standing knowledge of the MHO phenotype, there is still no expert consensus on the definition.^[8]^

CVDs and stroke are the leading cause of death, with several studies suggesting obesity as a risk factor.^[9,10]^ In a previous study, overweight and obese men revealed increased relative risk of stroke compared with men of normal weight, including ischemic and haemorrhagic stroke.^[10]^ Furthermore, lean hypertensive subject is suggested as another risk factor of CVDs in several previous studies.^[11,12]^ Metabolic syndrome (MetS) is also a suggested risk factor for cardiovascular and cerebrovascular disease. Previous studies suggest that MetS is not only an independent risk factor, but can also increase the odds ratio and hazard ratio of stroke.^[13]^ However, the relative risk of CVDs and stroke from obesity is relatively lower than that for other risk factors. Therefore, we assumed that obesity cannot be the sole predictor of CVDs and stroke incidence, and it is necessary to consider obesity in conjunction with metabolic components. Therefore, we retrospectively investigated the prevalence of CVDs (including ischemic heart disease [angina, myocardial infarction]) or stroke according to metabolic health with obese state.

## 2 Materials and methods

### 2.1 Subjects and data collection

In this study, we analyzed data of the 5th Korea National Health and Nutrition Examination Survey (KNHANES V-3). KNHANES V-3 is a cross-sectional survey conducted by the Division of Chronic Disease Surveillance of the Korean Center for Disease Control and Prevention.^[14]^ There are 4 parts: health interview survey, health behavior survey, health examination survey, and nutrition survey.^[15,16]^ A total of 8058 individuals were enrolled in KNHANES V-3. Among them, 4502 people >39 years of age were selected for this study. Subjects with missing data concerning MetS components and body mass index (BMI) were excluded, resulting in a total of 3695 subjects used for this analysis.

### 2.2 Subject classifications

The definition of obesity in Asian adults suggested by World Health Organization (WHO) is BMI ≥25 kg/m^2^.^[17]^ For this study, we defined BMI cut-offs for normal weight, overweight, and obese designations as <23 kg/m^2^, 23–24.9 kg/m^2^, and ≥25 kg/m^2^, respectively. Because, study subjects in this study were East Asian people. There are 5 components in MetS: (i) systolic/diastolic blood pressure ≥130/85 mmHg, (ii) serum fasting triglycerides ≥150 mg/dL, (iii) high-density lipoprotein (HDL) <40 mg/dL for men and <50 mg/dL for women, (iv) serum fasting glucose ≥100 mg/dL, and (v) waist circumference ≥90 cm for men and ≥85 cm for women.^[18]^ The study population was divided into 6 groups: healthy (presenting none of the 5 MetS components) normal weight, healthy overweight, healthy obese, unhealthy (2 or more MetS components) normal weight, unhealthy overweight, and unhealthy obese. Until these days, there is no obvious definition of metabolically healthy and unhealthy. Therefore, we followed previous studies defined metabolically unhealthy as who has 2 or more metabolic syndrome components regardless of parameters.^[19,20]^

### 2.3 Measurement of laboratory and anthropometric parameters

Fasting blood samples were collected from subjects who had kept empty for at least 8 hours. Examiners centrifuged and refrigerated the blood samples at the examination site. These blood samples were transferred by iceboxes to the Seoul central laboratory within the day. In the central laboratory, a Hitachi Automatic Analyzer 7600 (Hitachi, Japan) and an HLC-723G7 (Tosoh, Japan) were used to measure serum glucose, total cholesterol, triglyceride, HDL, and hemoglobin A1c (HbA1c).

Examiners measured waist circumference at the narrowest part between the lower margins of the rib cage and the iliac crest. After the subjects were rest in sitting position for 10 minutes, blood pressure was measured. Three blood pressures recorded with 5 minutes interval.

### 2.4 Health-related factors and medical histories

Education status, socioeconomic status, vitamin or mineral supplement intake, and prescribed medications were concerned in health interview survey. Alcohol consumption was assessed by categorizing subjects into 2 groups: those who never drink or current consumers. Subjects who consumed <1 glass of alcohol in the past month were also categorized as those who never drink. Individuals were classified into 3 groups by smoking status: never smoke, ex-smokers, and current smokers. The physical activity level was determined by the frequency (day/week) of moderate physical exercise accompanied by >10-minute mild dyspnea. CVDs and stroke was defined by previous medical diagnosis.

### 2.5 Statistical analysis

The KNHANES V-3 database sample used in this study was extracted by stratified, clustered, and systematic sampling. When analyzing statistics, we considered strata, clusters, and weights. The SURVEYMEANS procedure was used in the average calculation, whereas the SURVEYREG procedure and the SURVEYFREQ procedure were used in association analysis for continuous and categorical variables, respectively.

To assess differences among groups, the continuous variables and categorical variables were analyzed by linear regression analysis using the SURVEYREG procedure and the Rao–Scott chi-square test of SURVEYFREQ procedure. All statistical analyses were performed using SAS version 9.2 (SAS Institute, US, Cary, NC) and significance was defined as *P* < 0.05.

The study protocol conforms to the ethical guidelines of the 1975 Declaration of Helsinki as reflected in a priori approval by the Korea Centers for Disease Control and Prevention institutional review board in Korea (No. 2012-01EXP-01-2C).

## 3 Results

### 3.1 Baseline characteristics

Baseline characteristics of the 3695 study subjects (aged 40–85 years) with 5 MetS components are presented in Table 1. In total, 1726 (46.6%) subjects were categorized as metabolically healthy and 1969 (53.2%) were metabolically unhealthy. Among the subjects, 276 (7.5%) were in the MHO group and 989 (26.7%) were categorized as metabolically unhealthy and obese.

**Table 1:**
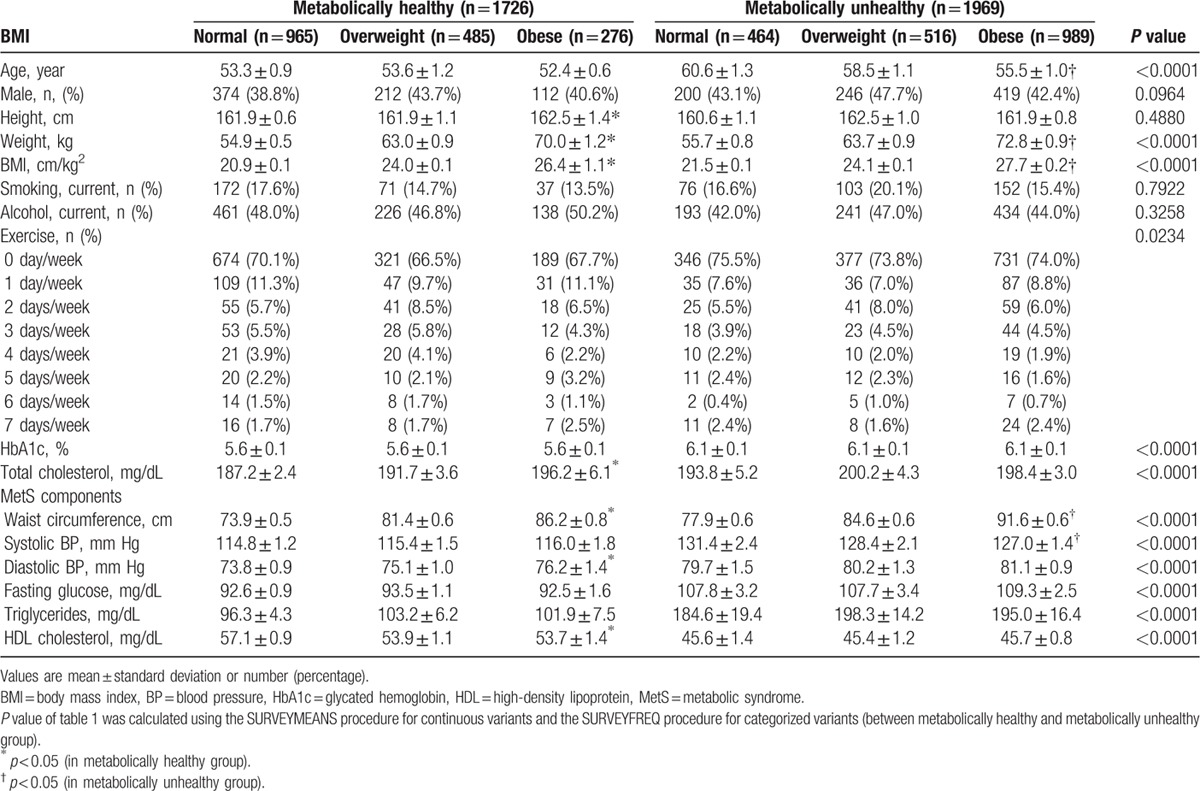
Baseline characteristics.

### 3.2 Comparison of CVDs or stroke prevalence

Of subjects in the healthy group, 76 (4.4%) were CVDs or stroke patients, whereas there were 170 (8.6%) CVDs or stroke patients in the unhealthy group (Table 2). There was a significant difference between the 2 groups in the prevalence of stroke (*P* < 0.001; Table 2). In the healthy group, there were 35 (3.6%), 26 (5.4%), and 15 (5.4%) CVDs or stroke patients in each of the subgroups (normal, overweight, and obese, respectively) divided by BMI status (Table 3). However, the prevalence of stroke did not reveal statistically significant differences among the 3 healthy subgroups (*P* = 0.4072; Table 3). In the unhealthy group, there were 43 (9.2%), 52 (10.0%), and 75 (7.6%) CVDs or stroke patients in each of the subgroups (normal, overweight, and obese, respectively) divided by BMI status (Table 3). There was also no significant difference in the CVDs or stroke prevalence of subjects in the unhealthy subgroups (*P* = 0.3798; Table 3).

**Table 2:**
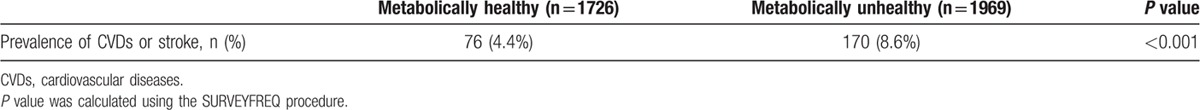
Comparison of cardiovascular disease and stroke prevalence according to the 2 groups by metabolic health status.

**Table 3:**
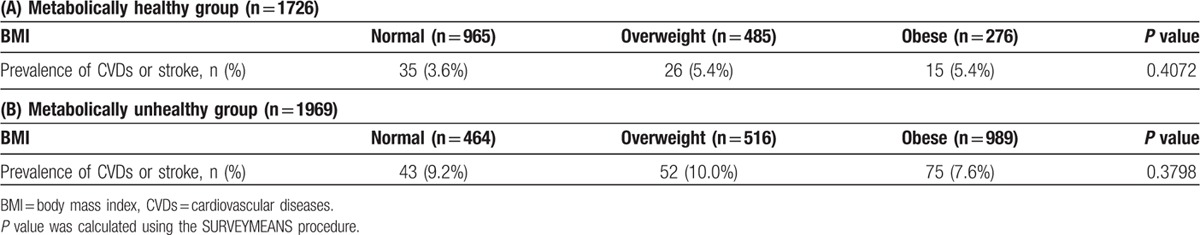
Comparison of cardiovascular disease and stroke prevalence according to the 3 subgroups by BMI status in the metabolically healthy group (A) and metabolically unhealthy group (B).

## 4 Discussion

The purpose of the present study was to reveal the relationship between the MHO phenotype and the prevalence of CVDs or stroke. To our knowledge, no previous study has compared CVDs or stroke risk in the MHO phenotype by class of obesity. As mentioned previously, the definition and prevalence of MHO are quite heterogeneous. A systematic review showed a large variation in the MHO prevalence, from 6% to 75%.^[21]^ In our study, overall prevalence of MHO was 7.5%.

In this study, subjects in metabolically healthy groups showed significantly lower prevalence of CVDs or stroke than those in metabolically unhealthy groups. Furthermore, there was no statistically significant difference between the prevalence of CVDs or stroke according to BMI in the metabolically healthy group. Therefore, we could confirm that MHO people did not reveal higher CVDs or stroke prevalence than metabolically healthy normal weight and overweight people. However, the metabolically unhealthy group revealed higher prevalence of CVDs or stroke regardless of obese state.

There are several suggested mechanisms that can explain the results of the present study. First, we suggest that MHO subjects who exhibit less amounts of visceral adipose tissue or ectopic fat and more physically active may experience a lower prevalence of stroke compared with metabolically unhealthy subjects. Sims^[22]^ highlighted visceral adiposity and insulin resistance as key factors of MHO. Metabolically healthy obese individuals had less amounts of visceral adipose tissue and liver fat, in contrast metabolically unhealthy nonobese individuals had a tendency to have plenty of visceral or ectopic fat.^[23–25]^ A previous study mentioned the “hypertriglyceridemic waist,” which means a phenotype who had an excessive visceral adipose tissue, increased waist circumference, and elevated plasma triglyceride levels.^[26]^ Another study also suggested that the percentage of visceral adipose tissue has to be regarded as a risk factor for stroke, small-artery occlusion, and large artery atherosclerosis type.^[27]^

Behavior and lifestyle factors may also play a substantial role. Physical activity increased the odds of presenting with the MHO phenotype among obese subjects.^[28]^ Increased sitting behavior could be associated with increased risk of cardiovascular events, diabetes mellitus, and all-cause mortality.^[29]^ At the same time, participating in physical activities can lower blood pressure, help control blood sugar levels, and contribute to weight loss. Therefore, a guideline from the American Heart Association and the American Stroke Association stroke council recommended walking for >30 minutes/d as a means of primary stroke prevention.^[30]^ In the present study, metabolically healthy subjects exercised more than metabolically unhealthy subjects (*P* = 0.0234; Table 1).

Furthermore, MetS and its components also contributed to our results. Several previous studies^[13]^ suggested that MetS is an independent risk factor of CVDs and stroke, and odds ratios and hazard ratios can be elevated by this condition. Components of MetS such as insulin resistance, dyslipidaemia, and hypertension are also independent risk factors of CVDs and stroke.^[30]^ Among these major risk factors, obesity showed the lowest influence on CVDs and stroke prevalence. One study determined the population attributable risk and relative risk (RR) factor of major risk factors for ischemic stroke in the Korean population.^[31]^ In the study, the RR of obesity (men, 1.2–1.9; women, 1.2) was lower than for other major risk factors such as hypertension (men, 2–2.8; women, 2–2.8), diabetes (men, 1.4–2.1; women, 1.72–2.3), hypercholesterolemia (men, 1.46–1.7; women, 1.46–1.7), atrial fibrillation (men, 4.3; women, 6.9), ischemic heart disease history (men, 1.7; women, 1.6), and stroke history (men, 3.1; women, 3.1). Therefore, we postulate that the synergetic effects of these risk factors, which revealed higher RR than obesity in metabolically unhealthy subjects, places them at prevalence of stroke rather than subjects in the metabolically healthy group.

Our study has a few limitations. We could not use a cohort for sampling purposes. Because this was a cross-sectional study, we were only able to estimate the prevalence of CVDs or stroke. Therefore, the results of this study cannot explain the causality between CVDs or stroke incidence and subjects exhibiting the MHO phenotype. Further studies are necessary to examine the differences between MHO and metabolically unhealthy obese phenotypes. One study in mice found that extremely obese mice with a mutation in the Brd2 gene are protected from developing type 2 diabetes mellitus.^[32]^ Therefore, we assume that future studies should concern the genetic and lifestyle which could be factors for development and improvement of metabolic conditions. Obesity assessed only by BMI cannot properly estimate the risk of CVDs or stroke, especially in East Asian populations including Koreans. Therefore, a paradigm shift of obesity is needed.^[5]^
